# Differentiating Colorectal Carcinoma From Diverticulitis With Computerised Tomography: Does Every Patient Need Follow-Up Colonoscopy After an Episode of Acute Diverticulitis?

**DOI:** 10.7759/cureus.12027

**Published:** 2020-12-11

**Authors:** Sandeep Singh, James Shuttleworth, Upekha Alagoda, Alice Giucca, Anna Heylen, Nick Browning, Haytham Sumrien

**Affiliations:** 1 General and Colorectal Surgery, North Bristol NHS Trust, Bristol, GBR

**Keywords:** diverticulitis, carcinoma, ct, endoscopy, polyp

## Abstract

Purpose

To correlate computerised tomography (CT) and endoscopic follow-up (FU) in differentiating presentations of acute diverticulitis (AD) and colorectal carcinoma (CRC).

Methods

Patient’s discharge summaries between April 2018 and September 2019, stating AD under-diagnosis were retrieved. Admission details, CT reports, endoscopic findings and histopathology results were retrospectively collected from prospectively maintained data.

Results

In our study period of 17 months, we identified 150 patients with an admission diagnosis of AD. In total, 134 patients had a CT confirmed diagnosis of AD; 61% had uncomplicated acute diverticulitis (UAD) and 39% complicated acute diverticulitis (CAD). The mean age of the patients was 64 years, and 59% were female. Of the 134, 15 patients were excluded, and 119 with AD were discharged with a plan to have FU endoscopy. Overall, 75% of the patients managed to undergo complete endoscopic investigation, 4% had incomplete endoscopy, and 21% failed to attend endoscopy. Follow-up (FU) endoscopic investigation found polyps in 20 patients; seven were reported as tubular adenomas with low-grade dysplasia and 10 as non-concerning hyperplastic or sessile polyps. One was inflammatory, and two were malignant. CT scans for two patients with malignant polyps were reported as CAD with suspicion of sigmoid cancer in one and right-sided perforated diverticulitis in the other. Both patients were female aged over 60 years.

Conclusions

This study demonstrates that a selective approach is more appropriate for endoscopic FU after an episode of AD. Risk stratification is required to allocate FU endoscopic investigation for patients at high risk for CRC.

## Introduction

In the United Kingdom, 50% of the population of 50 years is affected by diverticulosis; the prevalence increases with age [1]. The majority of those affected remain asymptomatic, though a proportion suffers acute diverticulitis (AD) which is described as an inflammation of diverticula, secondary to faecalith obstruction with localised mucosal inflammation [2]. AD is challenging to diagnose with acumen alone accurately. A combination of symptoms, inflammatory markers, and response to treatment guide suspicion, though ultrasound and computerised tomography (CT) are superior for diagnostic purposes [3]. CT imaging of the abdomen and pelvis is the gold standard in radiological diagnosis due to its high sensitivity and specificity [4,5].

Despite clinical presentation and a suggestive CT, the occasional lack of specific symptoms, examination and radiological findings prevent diagnostic confirmation of AD. Radiological findings such as vascular engorgement or mesenteric root fluid have been shown to have high specificity but low sensitivity for sigmoid diverticulitis [6]. Similarly, sections of colonic inflammation of >10cm are considered more specific for diverticulitis, whereas sections of <5cm or peri-colonic lymphadenopathy are more concerning for underlying carcinoma [7]. However, inflammatory bowel disease, infective colitis or malignancy may cause radiological changes similar to those observed in diverticulitis; inflammatory fat stranding, mural thickening or colonic obstruction [8]. The Royal College of Surgeons (RCS) recommended in 2014 that all patients require investigation of the colonic lumen by endoscopy, barium enema or CT colonography (CTC) after an acute episode has resolved, which is typically performed after 6-8 weeks [9]. This stance is supported by the Association of Coloproctology in Great Britain and Ireland (ACPGBI) who similarly state “Barium enema or colonoscopy after the resolution of the acute episode is required to rule out another diagnosis” [10].

In the last decade, with the increasing diagnostic accuracy of CT, the necessity of follow up (FU) endoscopy to exclude colorectal carcinoma (CRC) has been questioned with no clear guidelines. We conducted this retrospective study to build upon previous work and assess whether CT findings alone are enough to diagnose AD and accurately exclude CRC.

## Materials and methods

This retrospective study was conducted over 17 months (25/04/2018 to 25/09/2019) at our unit. Sequential surgical records of patients treated for acute diverticulitis were reviewed. Patients were identified by confirmed diagnoses or diagnostic suspicion of diverticulitis from discharge summaries stored in our hospital's secured database, Integrated Clinical Environment (ICE).

Admission details, CT reports, endoscopy findings and histopathology results were retrospectively collected from prospectively maintained data. CT reports from Synapse (Picture Archiving and Communication System - PACS) were coordinated with admissions, and assessment of diagnostic confidence was made. Radiology consultants reported all scans.

CT scan requests were correlated with Endoweb, an endoscopy reporting software. Cross-sectional imaging was compared with direct visualisation to evaluate the benefits and deficiencies of both modalities in the diagnosis of AD and CRC. Subjects who deceased before discharge or during follow-up and who underwent inpatient surgical intervention like Hartmann's procedure were excluded from FU, as were those where who were too frail for invasive intervention. A further group of patients who had a recent endoscopy or CTC were not included in our study.

The study was carried out within the framework of our institute, and the protocol was approved by The Patient Safety, Assurance and Audit Service (PSAAS) committee of North Bristol NHS Trust to proceed as an observational study. Informed consent was waived, and no identifying information (such as names, images and identifying data) is included in the article.

Statistical Analysis

For the descriptive analysis, mean was used for normally distributed data. For comparing numerical data, an unpaired t-test was used when dealing with normally distributed data and Mann-Whitney U test for non-parametric data.

## Results

We identified 150 patients who were diagnosed with AD during our study period of 17 months. Interestingly, 40 (27%) were known to have a diverticular disease (DD). CT scan was performed for 145 of them to confirm the diagnosis of AD, and five patients were diagnosed clinically without CT. Table 1 highlights the findings of CT scans.

**Table 1:** Findings of CT scan (n=145) CT- computerised tomography; UAD- uncomplicated acute diverticulitis; CAD- complicated acute diverticulitis;

CT Findings	Number of patients
UAD	82 (57%)
CAD	52 (35%)
Colitis	6 (4%)
Colon Cancer	1 (1%)
Diverticulosis only	3 (2%)
Normal CT	1 (1%)

In total, 134 patients had a CT confirmed diagnosis of AD; 61% had uncomplicated acute diverticulitis (UAD) and 39% complicated acute diverticulitis (CAD). The mean age of the patients was 64 years. The ratio of male to female patients was 41% and 59%.

**Figure 1:**
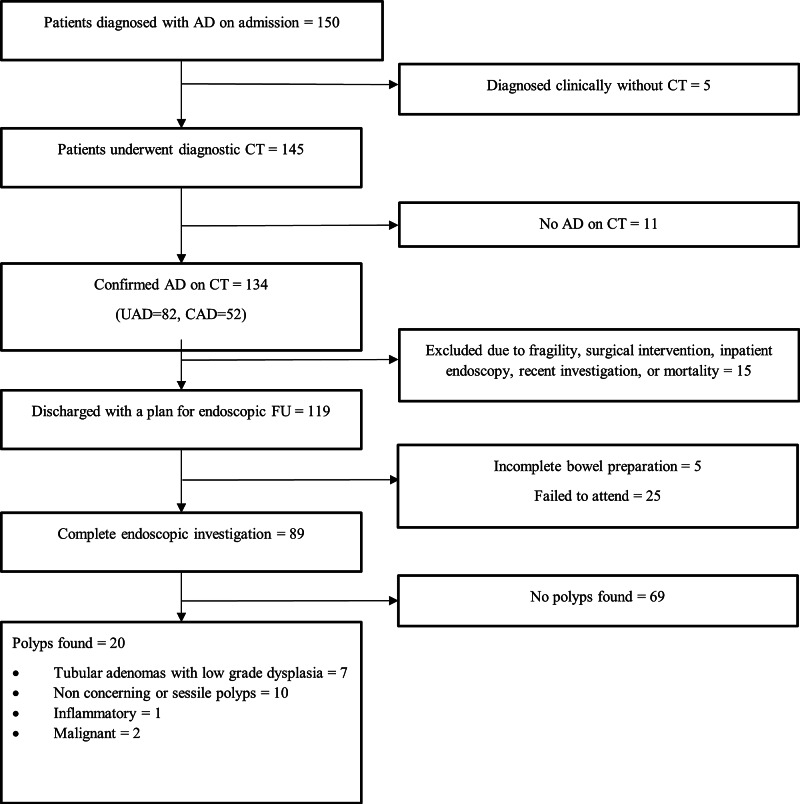
Inclusion and exclusion flowchart AD- Acute diverticulitis, CT- Computerised tomography, UAD- Uncomplicated acute diverticulitis, CAD- Complicated acute diverticulitis

**Figure 2:**
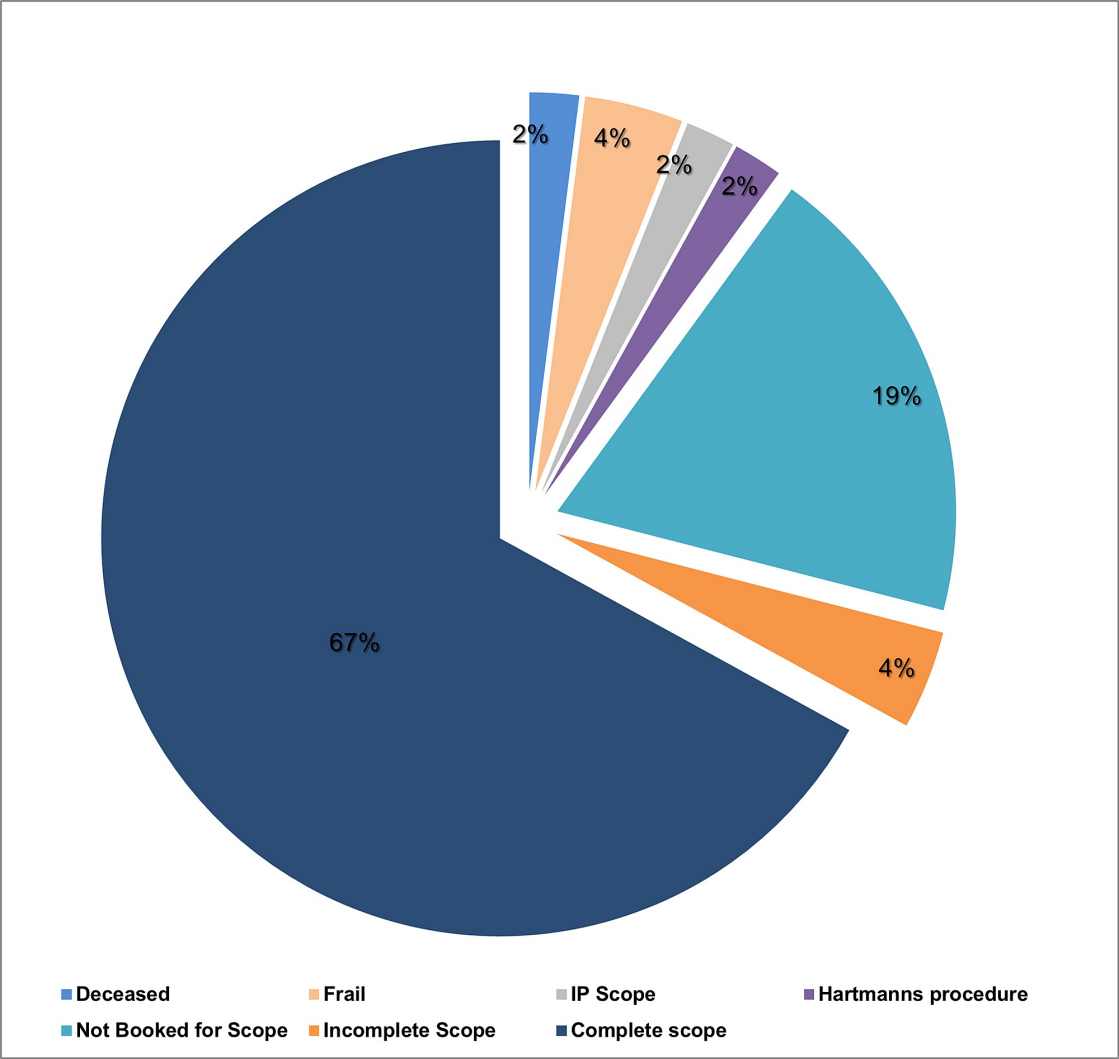
Outcome of CT proven acute diverticulitis (n=134)

Of the 134, 15 were excluded because of frailty, emergency surgical intervention like Hartmann's procedure, inpatient (IP) endoscopy, recent endoscopic evidence of diverticulosis and mortality (Table 2). In total, 119 patients with AD were discharged with a plan to have FU endoscopy. Several patients who underwent complete endoscopic investigation in the form of either flexible sigmoidoscopy or colonoscopy were 89 (75%), 5 (4%) had incomplete endoscopy due to poor bowel preparation or intolerance, and 25 (21%) failed to attend endoscopy (Figure 1 & 2).

**Table 2:** Excluded patients (n=15)

Reason for Exclusion	Number of patients
Deceased (Inpatient)	2
Deceased after discharge- grade 3 subarachnoid haemorrhage from an internal carotid aneurysm	1
Hartmann’s procedure	3 (2 as inpatient, 1 elective)
Frail- Not fit for any intervention or investigation	6
IP flexible sigmoidoscopy	3

FU endoscopic investigation found that 20 patients with polyps; 7 (9%) were reported as tubular adenomas with low-grade dysplasia and 10 (11%) as non-concerning hyperplastic or sessile polyps. One was inflammatory, and 2 (2%) were malignant (Table 3 & 4). Interestingly, CT scan for two patients with malignant polyps was reported as CAD with suspicion of sigmoid cancer in one and right-sided perforated diverticulitis in other (Table 5).

**Table 3:** Endoscopy findings (n=89)

Endoscopic findings	Number of patients
Normal	9 (10%)
Mild sigmoid inflammation/ patches of erythema	4 (4%)
Diverticular disease	56 (63%)
Polyp	20 (23%)

**Table 4:** Polyps at FU Endoscopic investigation (n=20) TA= Tubular adenoma, TV/VA= Tubulo-villous adenoma, SS= Sessile serrated, HP= Hyperplastic polyp, UAD= Uncomplicated acute diverticulitis, CAD=Complicated acute diverticulitis

Polyps (n=20)	Ascending/Caecum	Transverse	Descending	Sigmoid	Rectum
TA (n=7)			2 (2 UAD)	3 (1 UAD) (2 CAD)	2 (2 CAD)
SS (n=2)		1 (UAD)		1 (CAD)	
HP (n=8)			1 (CAD)	4 (1 UAD) (3 CAD)	3 (3 UAD)
Inflammatory (n=1)				1 (CAD)	
Malignant (n=2)	1 (CAD)			1 (CAD)	

## Discussion

CT scan is a widely used tool to assist diagnosis of AD, but radiological features of AD and CRC could be incorrectly interpreted. Despite certain findings being more specific to AD, there is overlap with features seen in CRC. Traditionally, FU colonoscopy is recommended by different colorectal societies after AD to rule out underlying malignancy.

However, in the last decade, there has been a substantive improvement in image quality and diagnostic accuracy of CT scans [11], questioning whether FU endoscopic investigation is required in patients with AD. The main aim of this study was to determine whether FU endoscopy for all patients with CT proven AD adds clinical value in ruling out an alternative diagnosis, particularly CRC, or should it be reserved for a selected subgroup of patients alone. Furthermore, a reduction in procedures would be cost-effective for NHS and less distressing and time consuming for patients. 

In our study, CT scan reports were mostly concordant with FU endoscopic findings. On luminal evaluation, 80% of the patients had a diverticular disease, and 2% (two patients) were found to have a malignant polyp. Scans of both patients with latterly proven malignant changes were reported as CAD, with suspicion of sigmoid cancer in one and right-sided perforated diverticulitis in the other, which independently warrant further investigation. AD, either uncomplicated or complicated, is not considered an independent risk factor for CRC. However, recent studies have observed a significantly higher incidence of colorectal carcinoma, particularly though not exclusively in CAD versus the general population [12]. 

Among the 89 patients who successfully underwent FU luminal evaluation, malignancy was diagnosed in two patients. The overall prevalence of malignancy among patients with CT proven AD was 1.5%. In literature, there are many systematic reviews and meta-analyses which have demonstrated similar findings to ours and concluded that routine colonoscopy after an episode of AD is not necessary [13-17]. In these studies, the prevalence of CRC among patients with radiologically diagnosed AD was less than 2%. However, few of these studies excluded patients with CAD. Our results showed that CRC prevalence was found to be higher in patients with CAD, thus making a point that only CAD requires FU endoscopic assessment. Also, both patients with malignant polyps were female with age > 60 years (Table 5).

**Table 5:** Comparing CT and FU polyp detection rate with age groups DD- Diverticular disease, UAD- Uncomplicated acute diverticulitis, CAD- Complicated acute diverticulitis

Age Groups (n=89)	Sex Female =47 Male = 42		Known DD (n=17)	CT UAD (n=53)	CT CAD (n=36)	Endoscopic Findings = Colonic polyp (n=20)	Histopathology= confirmed neoplasia (n=2)
31-50 years (n=17)	F	5	0	5	0	0	0
	M	12	3	6	6	4	0
51-65 years (n=37)	F	17	2	12	5	4	2
	M	20	2	8	12	8	0
66-80 years (n=25)	F	17	5	11	6	3	0
	M	8	1	5	3	1	0
>80 years (n=10)	F	8	3	5	3	0	0
	M	2	1	1	1	0	0

Since 2014, the year in which the guidelines regarding endoscopic FU were published, massive improvements have been made in the quality of CT scanners to provide high-resolution images that may allow a radiologist to more easily distinguish malignancy from inflammatory changes. The physical scanning process is a mature technology; however, better display programmes allow images to be viewed with greater resolution [18].

This study shows that a selective approach based on patients with certain risk factors such as CAD, female gender, and age (>60) may be beneficial to allocate resources and minimise patient distress appropriately. However, future work is required with a larger study cohort to evaluate this preliminary finding and a proposed algorithm for diagnostic accuracy and precision. Until this is the case, we recommend routine FU luminal evaluation to differentiate AD from CRC.

The strength of this study lies in its design and primary end-point question, which lays the foundation of a more extensive study in the future. Its weakness is that it is retrospective, small cohort and reliance on patients recorded data. Accordingly, we see a particular need for further prospective research on FU endoscopic investigation after an episode of AD to establish the selective criteria for risk stratification.

## Conclusions

Routine endoscopic evaluation after an episode of CT proven AD does not appear to be necessary to differentiate AD from CRC. This study justifies the need to develop a risk stratification algorithm based on CT findings, gender and age. However, further research is required with a more extensive study cohort to establish selective criteria.
